# Optimization of antimicrobial nanocomposite films based on carboxymethyl cellulose incorporating chitosan nanofibers and Guggul gum polysaccharide

**DOI:** 10.1038/s41598-024-64528-0

**Published:** 2024-06-13

**Authors:** Hamed Mohammadi, Mohammadreza Rezaeigolestani, Mohammad Mohsenzadeh

**Affiliations:** Department of Food Hygiene and Aquaculture, Faculty of Veterinary Medicine, Ferdowsi University of Mashhad, Mashhad, Iran

**Keywords:** CMC nanocomposites, Chitosan nanofiber, Guggul gum, RSM, Food packaging, Biopolymers, Nanocomposites, Polymer characterization

## Abstract

The present study utilized response surface methodology (RSM) to investigate the impact of varying concentrations of carboxymethyl cellulose (CMC: 0.75–1.75 wt%), *Commiphora mukul* polysaccharide (CMP: 0–1 wt%), and Chitosan Nanofiber (CHNF: 0–1 wt%) on the physical and antimicrobial characteristics of nanocomposite films based on CMC. The optimization process aimed to enhance ultimate tensile strength (UTS), strain at break (SAB), and antibacterial activity, while minimizing water vapor permeability (WVP), solubility, swelling, moisture content, opacity, and total color difference (ΔE). The results revealed that both CMP and CHNF had a positive influence on reducing moisture content, WVP, and increasing UTS. However, higher concentrations of CMP and CHNF had a divergent effect on SAB, ΔE, and swelling. The incorporation of CMP led to increased opacity and solubility, while the inclusion of CHNF resulted in decreased opacity and solubility. Notably, only CHNF addition significantly improved the antibacterial properties of the films. By applying the optimization procedure utilizing RSM, the formulation containing CMC (1.5 wt%), CMP (0.25 wt%), and CHNF (0.75 wt%) demonstrated superior physical, mechanical, and antibacterial properties in the biodegradable film matrix. These findings highlight the potential of utilizing these components to enhance the performance of CMC-based nanocomposite films.

## Introduction

The use of plastic films and coatings has revolutionized the food industry by improving the quality and extending the shelf life of fresh and processed foods^1^. However, the environmental concerns associated with synthetic packaging waste have led to the development of new biodegradable alternatives^2^.

In this context, natural biopolymers have emerged as a promising solution. Cellulose derivatives, such as carboxymethyl cellulose (CMC), are particularly attractive due to their low cost and film-forming ability^3^. Studies have already demonstrated that cellulosic materials are nontoxic, hemostatic, non-allergic, biocompatible, and biodegradable^4^. Although CMC has shown excellent film-forming properties, its mechanical strength is often inadequate. To address this limitation, blending CMC with other polymers has been explored^5^.

Chitosan, a natural cationic polysaccharide derived from chitin, has gained attention as an edible coating material with antimicrobial properties^6^. Nanochitosan, in particular, exhibits enhanced antibacterial activity compared to regular chitosan^7^. By converting chitosan into chitosan nanofibers (CHNF), its utility as a reinforcement filler in nanocomposites for food-active packaging films can be further enhanced^8^.

Various studies have considered the use of nanofillers in CMC matrices. The results of those studies suggested that chitin and chitosan nanoparticles have a good potential to be used in CMC film while creating some positive changes in mechanical and water vapor barrier properties of the developed composites^2,3^. In addition, Many studies have demonstrated that the polysaccharides, when incorporated into the CMC-based films, could be positively effective in The physical and mechanical properties, color, transmittance, antioxidant, and antimicrobial activities^8,9^. Furthermore, the combination of natural polysaccharides has shown promising results in different fields. For instance, the use of chitosan and myrrh polysaccharide extract in cellulosic dressings demonstrated enhanced antibacterial performance^10^.

Another potential candidate for reinforcing the polymer matrix in nanocomposites is a highly branched polysaccharide extracted from Guggul gum, derived from the resinous exudates of certain *Commiphora* species.

The incorporation of gums into carbohydrate films, especially in the context of nanocomposites, holds significant importance in enhancing their mechanical and functional properties^11^. Gums, being natural polymers, offer several advantages such as renewability, biodegradability, and cost-effectiveness^12^. When integrated into carbohydrate matrices like carboxymethyl cellulose (CMC), gums act as reinforcing agents, improving the overall strength, flexibility, and stability of the resulting nanocomposites^11^.

Guggul gum is derived from the resinous exudates of certain *Commiphora* species, native to the Indian subcontinent, North Africa, and parts of Central Asia. Traditionally used in Ayurvedic medicine, Guggul gum has gained attention in various industrial applications due to its unique properties^13^. Guggul gum primarily consists of a highly branched polysaccharide. It contains bioactive compounds like guggulsterone, which exhibit anti-inflammatory and antioxidant properties. These compounds make Guggul gum not only valuable in traditional medicine but also intriguing for scientific research^14^. the resinous exudates of certain *Commiphora* species, being natural, are biodegradable, aligning with the growing need for eco-friendly materials in various industries, including packaging and biomedical applications^10^. Therefore, the highly branched polysaccharide structure of Guggul gum makes it an excellent candidate for reinforcing polymer matrices, enhancing the mechanical strength of nanocomposites.

Considering the need to improve the properties of CMC-based films, it is crucial to explore the simultaneous utilization of different ingredients and evaluate their potential influences. Response Surface Methodology (RSM) offers a valuable approach for optimizing analytical procedures, providing advantages over traditional one-variable-at-a-time optimization. By generating large amounts of information from a small number of experiments, RSM allows for the evaluation of interaction effects between variables on the response^15^. In this study, we employed RSM with a central composite design (CCD) to investigate the effects of CMC, CHNF, and Guggul gum concentrations on the physicochemical and antimicrobial properties of CMC-based biodegradable films. The ultimate goal was to achieve an optimized active nanocomposite formulation.

Through the optimization of CMC-based biodegradable nanocomposite films containing chitosan nanofibers and Guggul gum polysaccharides, this research aims to contribute to the development of environmentally friendly packaging materials that possess enhanced mechanical strength and antimicrobial properties.

## Materials and methods

### Materials

CMC (viscosity of 1% solution in water at 20 °C is 1500–3500 centipoises, with a pKa of 3.5, and molecular weight of 41,000 g/mol) and 99% pure glycerol were obtained from Merck, Germany. The CHNF was provided by Nano Novin Polymer Company in Iran. Guggul gum (Extraction of *Commiphora mukul*), which consists of oleo-gum resin, was purchased from a commercial market in Mashhad, Iran. Extraction of *Commiphora mukul* polysaccharide (CMP) was done according to the method of Alminderej, Fahad,^10^.

### Bacterial strains

Three well-known foodborne pathogens and yeast including *Staphylococcus aureus* PTCC 25923, *Salmonella enterica* PTCC 5177, *Escherichia coli* ATTC 12900, and *Saccharomyces cerevisiae* PTCC 1709 were selected for antimicrobial analysis, and they were obtained from the Persian Type Culture Collection of the Iranian Research Organization for Science and Technology. The microorganisms were cultured in Brain Heart Infusion Broth (BHI Broth) at 37 °C for 24 h prior to the experiment.

### Experimental design

In this study, a central composite design (CCD) of RSM was employed to investigate the effects of CMC concentration (X_1_) (0.75–1.75 wt%), CMP concentration (X_2_) (0–1 wt%), and CHNF concentration (X_3_) (0–1 wt%) on various properties of CMC-based biodegradable films. The dependent variables studied included solubility (%), swelling (%), moisture content (%), water vapor permeability (WVP, g/mhPa), ultimate tensile strength (UTS, MPa), strain at break (SAB, %), color difference (ΔE), thickness (mm), opacity, and antibacterial activity. The levels of the independent variables were determined based on previous studies, and actual variables were coded to facilitate multiple regression analysis. A total of 17 formulations were prepared according to the CCD, with runs 2, 5, and 7 at the center points used to determine the experimental error and data reproducibility. The complete design matrix of the experiments is presented in Table 1.

**Table 1:** Central composite design with independent variable for preparation of CMC-based films.

Run	Coded variables			Uncoded variables		
	X_1_	X_2_	X_3_	CMC^a^ (wt%)	CMP^b^ (wt%)	CHNF^c^ (wt%)
1	0.000	0.000	− 2.000	1.25	0.5	0
2	0.000	0.000	0.000	1.25	0.5	0.5
3	0.000	2.000	0.000	1.25	1	0.5
4	− 1.000	1.000	1.000	1	0.75	0.75
5	0.000	0.000	0.000	1.25	0.5	0.5
6	1.000	− 1.000	− 1.000	1.5	0.25	0.25
7	0.000	0.000	0.000	1.25	0.5	0.5
8	2.000	0.000	0.000	1.75	0.5	0.5
9	1.000	1.000	− 1.000	1.5	0.75	0.25
10	1.000	1.000	1.000	1.5	0.75	0.75
11	− 1.000	− 1.000	− 1.000	1	0.25	0.25
12	0.000	− 2.000	0.000	1.25	0	0.5
13	0.000	0.000	2.000	1.25	0.5	1
14	1.000	− 1.000	1.000	1.5	0.25	0.75
15	− 1.000	1.000	− 1.000	1	0.75	0.25
16	− 2.000	0.000	0.000	0.75	0.5	0.5
17	− 1.000	− 1.000	1.000	1	0.25	0.75

^a^CMC, Carboxymethyl cellulose. ^b^CMP, *Commiphora mukul* polysaccharide. ^c^CHNF, Chitosan nanofiber. X_1_: CMC concentration (0.75–1-75%); X_2_: CMP concentration (0–1%), and X_3_: CHNF concentration (0–1%).

### Preparation of the films

To prepare the CMC-based films, 0.75–1.75 g of CMC powder was mixed with 100 mL of distilled water under stirring at 50 °C for approximately 45 min. Glycerol (50% v/w based on CMC weight) was added as a plasticizer. Then, CMP (0–1 wt%) was added to the solution and stirred for 15 min at 50 °C. The CHNF (0–1 wt%) was mixed with the CMC/CMP solution, and the film solution was homogenized at 3000 rpm using an IKA T25-Digital Ultra Turrax (Stavfen, Germany). The resulting solution was poured into a circular plate with a diameter of 15 cm and a circular area of 177 cm^2^. The prepared films were dried in a hot air oven at 60 °C for 4 h, then placed in a plastic bag and stored in a desiccator in a digitally controlled humidity drying cabinet (25 °C, 50% relative humidity (RH)) until the experiments.

### Characterization of the films

#### Thickness

To determine the thickness of the film, a measurement technique was employed that involved randomly selecting five points on the film surface and measuring the distance between the top and bottom surfaces at each point using a digital micrometer manufactured by Mitutoyo (model no. 293–766, Tokyo, Japan). The average thickness of the film was then calculated based on these five measurements. This method was chosen for its accuracy and ease of use in measuring thin films^16^.

#### Water sensitivity of CMC-based film

##### Water solubility of the films

The solubility of the films was determined by immersing a known weight (W1) of the film in 50 mL of distilled water for 24 h at 25 °C. After 24 h, the swollen film was removed, and the remaining undissolved residue was dried in a hot air oven at 105 °C until a constant weight (W2) was achieved. The percentage of solubility was calculated using Eq. (1)^17^:

1 $$\% {\text{ Solubility }} = \, {{\left( {{\text{W1 }} - {\text{ W2}}} \right)} \mathord{\left/ {\vphantom {{\left( {{\text{W1 }} - {\text{ W2}}} \right)} {{\text{W1 }} \times { 1}00}}} \right. \kern-0pt} {{\text{W1 }} \times { 1}00}}$$

##### Swelling test

The swelling percentage of the films was determined by measuring the weight (W1) of the film before immersion in distilled water and the weight (W2) of the swollen film after 24 h immersion. The percentage of swelling was calculated using Eq. (2)^18^:

2 $${\text{Swelling }} = \, {{\left( {{\text{W2 }} - {\text{ W1}}} \right)} \mathord{\left/ {\vphantom {{\left( {{\text{W2 }} - {\text{ W1}}} \right)} {{\text{W1 }} \times { 1}00}}} \right. \kern-0pt} {{\text{W1 }} \times { 1}00}}$$

##### Water vapor permeability (WVP)

To determine the WVP, a gravimetric method was employed at a temperature of 25 °C using the ASTM (1995) standard^19^. Permeation cells with a diameter of 1 cm and a depth of 3.5 cm were used for the study. The specimens were equilibrated at a relative humidity (RH) of 65 ± 2% and a temperature of 25 ± 2 °C for 48 h prior to the test. Anhydrous silica gel (0% RH) weighing 2.5 g was placed in each permeation cell, which was then covered with the film sample. The permeation cell was subsequently placed in a desiccator containing a small beaker with saturated K_2_SO_4_ solution at the bottom, and a small amount of solid K2SO4 was left at the bottom of the saturated solution to maintain its saturation. The saturated K_2_SO_4_ solution in the desiccator provided a constant RH of 98% at 25 °C. The weight changes of the permeation cell were recorded every 8 h as a function of time. The slopes were determined by linear regression analysis (weight change vs. time), and the water vapor transmission rate (WVTR) was defined as the slope (g/h) divided by the transfer area (m^2^). Finally, the WVP (g/m h Pa) was calculated using Eq. (3).

3 $${\text{WVP }} = \, {{{\text{WVTR }} \times {\text{ X }}} \mathord{\left/ {\vphantom {{{\text{WVTR }} \times {\text{ X }}} {\left( {{\text{P }} \times \, \left( {{\text{R1 }} - {\text{ R2}}} \right)} \right)}}} \right. \kern-0pt} {\left( {{\text{P }} \times \, \left( {{\text{R1 }} - {\text{ R2}}} \right)} \right)}}$$

where P is the saturation vapor pressure of water (Pa) at the test temperature (25 °C), R1 is the RH in the desiccator, R2 denotes RH in the permeation cell, and X is the film thickness (m). All measurements were performed in triplicate.

##### Moisture content

To conduct this analysis, the empty capsules were placed in an oven at a temperature of 110 °C for one hour until they reached a constant weight. Next, film samples were cut into pieces measuring 3 cm × 1 cm and placed inside the capsules. The combined weight of the film and capsule was recorded, and then the capsules were returned to the oven at 110 °C until they reached a constant weight. Once cooled in a desiccator, the weight of the dry film and capsule was measured to determine the dry sample weight. The moisture content of the films was calculated based on the wet weight using Eq. (4)^20^:

4 $${\text{Moisture content }}\left( \% \right) \, = \left[ {{{\left( {{\text{Initial weight }} - {\text{ Dry weight}}} \right)} \mathord{\left/ {\vphantom {{\left( {{\text{Initial weight }} - {\text{ Dry weight}}} \right)} {\text{Initial weight}}}} \right. \kern-0pt} {\text{Initial weight}}}} \right] \, \times { 1}00$$

#### Mechanical properties

The mechanical properties of the CMC-based films were evaluated by determining the ultimate tensile strength (UTS) and strain at break (SAB) using a TA.XTplus texture analyzer (Lloyd Instruments, London, England) The films were cut into dumbbell-shaped specimens with a gauge length of 40 mm and a width of 10 mm. The specimens were pulled at a crosshead speed of 5 mm/min until they broke. The UTS and SAB were calculated using Eq. (5)^21^:

5 $$\begin{gathered} {\text{UTS }}\left( {{\text{MPa}}} \right) \, = \, {{{\text{Maximum load at break }}\left( {\text{N}} \right)} \mathord{\left/ {\vphantom {{{\text{Maximum load at break }}\left( {\text{N}} \right)} {{\text{Cross}} - {\text{sectional area of the specimen }}\left( {{\text{mm}}^{{2}} } \right)}}} \right. \kern-0pt} {{\text{Cross}} - {\text{sectional area of the specimen }}\left( {{\text{mm}}^{{2}} } \right)}} \hfill \\ {\text{SAB }}\left( \% \right) \, = \, {{\left( {{\text{Final gauge length }} - {\text{ Initial gauge length}}} \right)} \mathord{\left/ {\vphantom {{\left( {{\text{Final gauge length }} - {\text{ Initial gauge length}}} \right)} {{\text{Initial gauge length }} \times { 1}00}}} \right. \kern-0pt} {{\text{Initial gauge length }} \times { 1}00}} \hfill \\ \end{gathered}$$

#### Color analysis

The color properties of the films were evaluated using a HunterLab ColorFlex EZ 45/0 TM color spectrophotometer (HunterLab Reston, VA, USA). The colorimeter was calibrated using a standard white plate (L* =  + 97.83, a* = − 0.43, b* =  + 1.98). The films were cut into 4 cm × 4 cm squares, and each sample was analyzed in triplicate. The L*, a*, and b* values were measured, and the color difference (ΔE) between the film and the standard white plate was calculated using equation Eq. (6)^22^:

6 $$\Delta {\text{E }} = \, \left[ {\left( {\Delta {\text{L}}*} \right)^{{2}} + \, \left( {\Delta {\text{a}}*} \right)^{{2}} + \, \left( {\Delta {\text{b}}*} \right)^{{2}} } \right]^{{{1}/{2}}}$$

where ΔL*, Δa*, and Δb* are the differences between the L*, a*, and b* values of the film and the standard white plate, respectively. The color difference (ΔE) was used to evaluate the color changes of the films due to the addition of CHNF and CMP. The smaller the ΔE value, the closer the film color was to that of the standard white plate.

#### Opacity properties

The visible light-blocking properties of the films were evaluated through a spectrophotometer (CAMSPEC M550, Germany) at a 600 nm wavelength. The films were cut into 4 × 1 cm strips and inserted into a transparent cell wall. To calibrate the device, an empty cell was employed. The opacity of the films was determined in Eq. (7), where T600 represents the transmission rate at 600 nm, and X denotes the thickness of the film (mm)^23^.

7 $${\text{Opacity }}\left( \% \right) \, = \, \left( {{\text{T6}}00 \, /{\text{ X}}} \right) \, \times { 1}00$$

#### Antimicrobial activity of films by disc diffusion method

The antibacterial properties of the films were evaluated using the agar disc diffusion method^2^. The edible films were cut into discs with a diameter of 6 mm and then placed onto Mueller Hinton agar (Merck, Darmstadt, Germany) plates. Before this, the plates had been seeded with an inoculum of approximately 105–106 CFU/mL of *S. enterica*, *S. cerevisiae*, *S. aureus* and, *E. coli* bacteria, in a volume of 0.1 mL. Following incubation at 37 °C for 24 h, the diameter of the growth inhibition zone surrounding the film discs was measured using a caliper from the center of the film. We performed each assay in triplicate, over two separate experimental runs.

### Verification and optimization procedures

To achieve the best film properties, including acceptable solubility, moisture content, swelling, water vapor permeability, opacity, ultimate tensile strength, stretchability, color properties, and antibacterial activity against *S. enterica*, *S. cerevisiae*, *S. aureus*, and *E. coli*, was utilized numerical and graphical optimization procedures to determine the optimal levels of three independent variables (CMC, CHNF, and CMP). Design-Expert software was used to conduct both numerical and graphical optimizations and then compared the experimental data with the predicted values obtained from the regression models to verify their validity and adequacy.

### Film morphology

The morphology of the films was examined utilizing a scanning electron microscope (SEM), specifically the LEO-1450 VP model (Zeiss, Oberkochen, Germany). In order to prepare the film samples for analysis, they were securely affixed to the specimen holder and subsequently subjected to a gold sputter-coating process with a thickness of 10 mm, all conducted under vacuum conditions. Subsequently, SEM images were captured to assess the film's structural characteristics^24^.

### Fourier-transform infrared (FTIR) spectroscopy

The molecular interactions among CMC, CMP, and CHNF molecules within the structure of the developed composites were assessed using a Bruker Equinox spectrometer (Bruker Banner Lane, Coventry, Bremen, Germany). For this purpose, five samples were prepared and analyzed, including:

(A) CMC: 1.25 wt% / CMP: 0.5 wt%, (B) CMC: 1.25 wt% / CMP: 0.5 wt% / CHNF: 0.5 wt%, (C) CMC: 1.75 wt% / CMP: 0.5 wt% / CHNF: 0.5 wt%, (D) CMC: 1 wt% / CMP: 0.75 wt% / CHNF: 0.25 wt%, (E) CMC: 1 wt% / CMP: 0.75 wt% / CHNF: 0.25 wt%.

The analyses were conducted in the spectral range of 4000 to 400 cm^−1^ with a resolution of 4 cm^−1^. Sixteen scans were averaged for each spectrum^24^.

### Design-Expert 13.00 computer software

The experimental design, regression analysis of the experimental data, and plotting of response surface plots were conducted using Design Expert version 13.0.5.0 (Stat-Ease, Inc., Minneapolis, USA) computer software. The analysis of variance (ANOVA) tables were generated to determine the effect and regression coefficients of individual linear, quadratic, and interaction terms.

### Ethics approval

The authors confirmed that the institutional committee and licensing committee approved the experiments, including all relevant details and that all experiments have been performed in accordance with specified named guidelines and regulations. All methods were in accordance with relevant institutional, national, and international guidelines and legislation.

## Results and discussion

### Optimization of CMC-based films

#### Fitting of response surface model

A full response surface model was created for each response variable by employing regression analysis, which took into account linear (X_1_, X_2_, X_3_), interaction (X_1_ X_2_, X_1_X_3_, X_2_X_3_), and quadratic terms (${\text{X}}_{1}^{2}$, ${\text{X}}_{2}^{2}$, ${\text{X}}_{3}^{2}$). The experimental data obtained from the response variables are presented in Table 2.

**Table 2:** Parameters and optimization values of CMC/CMP/CHNF composite films for each of responses separately.

Name	Goal	Lower limit	Upper limit	Optimized value	Desirability
CMC^a^	Is in range	1	1.5	1.5	1
CMP^b^	Is in range	0.25	0.75	0.25	1
CHNF^c^	Is in range	0.25	0.75	0.75	1
UTS^d^	Maximize	8.9976	27.4356	19.611	0.57
SAB^e^	Maximize	0	24.99	16.404	0.65
opacity	Minimize	0.49	2.49	0.53	0.97
WVP^f^	Minimize	0.12	9.91	7.372	0.25
ΔE^g^	Minimize	0.1345	103.775	18.795	0.81
Solubility	Minimize	53.67	80.57	60.784	0.73
MOISTURE	Minimize	15.23	28.45	25.403	0.23
Swelling	Minimize	148.59	791.94	349.210	0.68
Thickness	Minimize	0.0199	0.0971	0.026	0.91
*S. aureus* disc diffusion	Maximize	0	6	4.622	0.77
*E. coli* disc diffusion	Maximize	0	5	4.209	0.84
*S. entrica* disc diffusion	Maximize	0	3.4	2.835	0.83
*S. cerevisiae* disc diffusion	Maximize	0	8	6.126	0.76
Desirability Combined					0.66

^a^CMC, Carboxymethyl cellulose. ^b^CHNF, Chitosan nanofiber. ^c^CMP, *Commiphora mukul* polysaccharide. ^d^UTS, ultimate tensile strength. ^e^SAB, strain at break. ^f^WVP, water vapor permeability. ^g^ΔE, color difference.

To assess the adequacy of the model, five parameters: the model itself, the F value, the lack of fit F value, the coefficient of determination R^2^, and the adjusted R^2^ were examined. Additionally, three different tests: lack of fit tests, model summary statistics, and coefficient of variation (CV) were performed.

All RSM data was obtained using Design-Expert. For each response, the software displayed F and P values, R^2^, adj-R^2^, and PRESS of the models. The best model with a significant F value, the highest value of R^2^ and adj-R^2^, and the lowest level of PRESS were then selected. The selection of the best model based on F value, R^2^, adj-R^2^, and PRESS ensures the robustness and reliability of the predictive models, providing confidence in their ability to accurately describe the experimental data. The selection of appropriate models for each response variable ensures that the model adequately captures the relationships between the independent variables and the responses. The use of both linear and quadratic models highlights the complex nature of the interactions between the variables and their impact on film properties.

Table 3 indicates that all the models obtained were statistically significant (P < 0.01) at a 99% confidence level. The quadratic model suggested for UTS, SAB, ΔE, solubility, swelling, and thickness. Additionally, the 2Fl model was suggested for opacity. Moreover, the linear model suggested moisture and the responses of *S. aureus*, *S. enterica*, *S. cerevisiae*, and *E. coli* to disk diffusion. And finally, both the linear and quadratic models were suggested for WVP. Furthermore, the coefficient of variation (CV) was less than 20% for all cases except for the responses to bacterial disk diffusion (Table 3). The coefficient of variation (CV) provides insights into the variability of the data. In our study, the CV was less than 20% for all cases except for the responses to bacterial disk diffusion. The higher CV in bacterial disk diffusion may be attributed to the inherent variability in biological assays, including variations in bacterial growth conditions, testing conditions, environmental factors, and measurement techniques.

**Table 3:** ANOVA and statistics values of CMC/ CMP/ CHNF composite films for each of responses separately after eliminating non-significant factors.

	Std. Dev	C.V. %	R^2^	Adjusted R^2^	Predicted R^2^	PRESS	P-values of Lack of Fit	F-values of Model	P-values of Model	Suggested Model
UTS^a^	0.8264	4.73	0.9853	0.9786	0.9312	35.20	0.296	147.61	< 0.0001	Quadratic
SAB^b^	1.14	12.20	0.9867	0.9787	0.9431	55.79	0.1101	123.79	< 0.0001	Quadratic
opacity	0.0502	3.04	0.9947	0.9929	0.9904	0.0546	0.9372	557.80	< 0.0001	2FI
WVP^c^	0.9485	17.56	0.9539	0.9180	0.7765	39.22	0.2044	26.58	< 0.0001	Quadratic
ΔE^d^	3.86	8.59	0.9932	0.9879	0.9683	623.19	0.1014	187.70	< 0.0001	Quadratic
solubility	0.8821	1.38	0.9938	0.9890	0.9720	31.81	47.37	207.32	< 0.0001	Quadratic
Moisture	1.28	5.95	0.9050	0.8830	0.8269	38.81	.0.0981	41.27	< 0.0001	Linear
Swelling	18.21	4.52	0.9908	0.9877	0.9564	18,823.83	0.2884	322.74	< 0.0001	Quadratic
Thickness	0.0021	3.45	0.9925	0.9900	0.9697	0.0002	0.1425	397.81	< 0.0001	Quadratic
*S. aureus*										
disc diffusion	0.7820	28.47	0.8598	0.8505	0.8153	12.08	0.0598	91.99	< 0.0001	Linear
*E . coli*										
disc diffusion	0.7005	27.38	0.8554	0.8458	0.8014	10.12	0.0741	88.76	< 0.0001	Linear
*S. entrica*										
disc diffusion	0.5019	28.92	0.8367	0.8258	0.7823	5.04	0.0776	76.85	< 0.0001	Linear
*S. cerevisiae*										
disc diffusion	0.7532	21.06	0.9244	0.9193	0.8845	13.01	0.0595	183.37	< 0.0001	Linear

^a^UTS, ultimate tensile strength. ^b^SAB, strain at break. ^c^WVP, water vapor permeability. ^d^ΔE, color difference.

In order to construct the response surface models, any terms that were found to be insignificant (P > 0.05) were removed. The resulting regression equations, in terms of coded factors, for predicting the impacts of CMC, CHNF, and CMP concentrations on the responses after eliminating non-significant factors, were expressed using polynomial equations (Eqs. 8–19).

In this research, the coefficient of determination (R^2^) values for solubility (%), swelling (%), WVP, and moisture (%) were determined to be 0.9938, 0.9908, 0.9539, and 0.9050, respectively. The corresponding adjusted R^2^ values were 0.9890, 0.9877, 0.9180, and 0.8830 (Table 3). Therefore, the quadratic models were significant (P < 0.05) for these dependent variables except for the moisture (%). The final regression equations for water sensitivity parameters are presented in the following equations:

8 $$Solubility\;(\% ) = 62.5611 + 6.37875X1 + 4.51625X2 - 2.4075X3 + 1.19X1X2 - 1.56X1X3 + 1.10569X_{1}^{2} + 0.411941X_{2}^{2}$$

9 $$Swelling\;(\% ) = 482.23 + 45.335X1 + 147.37X2 + 47.2488X3 + 21.7658X_{2}^{2}$$

10 $$WVP\,(g/mhPa) = 8.27 + 0.3056X1 - 2.80X2 - 0.2959X3 - 0.6613X1X3 - 0.9013X_{1}^{2} - 0.9363X_{2}^{2} - 1.21X_{3}^{2}$$

11 $${\text{Moisture }}\left( \% \right) \, = { 21}.{5112 } + { 3}.{17}0{\text{63X}}_{{1}} - { 1}.{\text{44813X}}_{{2}} - \, 0.{\text{726875X}}_{{3}}$$

As indicated in Table 3, the quadratic models for UTS and SAB yielded R^2^ coefficients of 0.9853 and 0.9867, respectively, demonstrating the effectiveness of the proposed models for each characteristic. The significance of the quadratic term coefficients in UTS and SAB was confirmed by very small p-values (p < 0.01), as shown in Table 4. However, the mutual interaction coefficients (X_1_X_2_, X_1_X_3_, X_2_X_3_) were found to be insignificant (p > 0.05) in the case of UTS. The final regression equations for UTS and SAB can be expressed using the following equations:

12 $$UTS\;(MPa) = 15.6804 + 2.02171X1 + 3.55603X2 + 3.56937X3 + 1.01848X_{2}^{2} + 0.877029X_{3}^{2}$$

13 $$SAB\;(\% ) = 8.36887 + 2.60875X1 - 6.75625X2 - 2.3875X3 - 0.7975X1X2 - 0.785X1X3 + 1.04495X_{2}^{2}$$

**Table 4:** Coefficients table of CMC(A), CMP(B) and CHNF(C) composite films for each of the responses separately before eliminating non-significant factors.

Response	Intercept	A	B	C	AB	AC	BC	A^2^	B^2^	C^2^
UTS^a^	15.8709	**2.02171**	**3.55603**	**3.56937**	− 0.060575	− 0.279725	− 0.10955	− 0.113126	**0.973824**	**0.832374**
p-values		< 0.0001*	< 0.0001*	< 0.0001*	0.8640	0.4391	0.7574	0.6217	0.0030*	0.0067*
SAB^b^	8.64739	**2.60875**	− **6.75625**	− **2.3875**	− 0.7975	− 0.785	0.55	0.119674	**0.979674**	− 0.350326
p-values		< 0.0001*	< 0.0001*	< 0.0001*	0.0546	0.0576	0.1560	0.6072	0.0031*	0.1592
opacity	1.63391	− **0.300625**	**0.450625**	− 0.029375	**0.33875**	− 0.02875	− 0.00625	0.00923913	− 0.00326087	0.0104891
p-values		< 0.0001*	< 0.0001*	0.0570	< 0.0001*	0.1593	0.7421	0.4568	0.7891	0.4010
WVP^c^	8.26991	0.305625	− **2.80187**	− 0.295875	0.26125	− 0.66125	− 0.36625	− **0.901261**	− **0.936261**	− **1.21101**
p-values		0.2444	< 0.0001*	0.2583	0.4675	0.0930	0.3173	0.0044*	0.0036*	0.0009*
ΔE^d^	37.1496	− **16.8654**	**16.5308**	**23.4796**	**6.53811**	− **10.1241**	1.97093	**4.29054**	− 0.129182	**4.04967**
p-values		< 0.0001*	< 0.0001*	< 0.0001*	0.0019*	0.0001*	0.1885	0.0017*	0.8861	0.0023*
solubility	62.3296	**6.37875**	**4.51625**	− **2.4075**	**1.19**	− **1.56**	− 0.3125	**1.15995**	0.466196	0.137446
p-values		< 0.0001*	< 0.0001*	< 0.0001*	0.0079*	0.0019*	0.3660	0.0008*	0.0598	0.5295
Moisture	21.4839	**3.17063**	− **1.44813**	− **0.726875**	0.60875	0.69875	0.64375	0.151739	− 0.259511	0.136739
p-values		< 0.0001*	0.0009*	0.0271*	0.1431	0.1002	0.1247	0.5427	0.3102	0.5824
Swelling	390.187	**45.335**	**147.37**	**47.2487**	− 2.4925	− 4.785	1.8025	− 3.07533	**19.9009**	− 3.51408
p-values		< 0.0001*	< 0.0001*	< 0.0001*	0.7589	0.5594	0.8240	0.5594	0.0054*	0.5064
Thickness	0.0583174	− **0.0080125**	**0.0180625**	− 0.0001	**0.008125**	6.92953E-19	6.81255E-20	**0.00237717**	5.21739E-05	-9.78261E-05
p-values		< 0.0001*	< 0.0001*	0.8871	< 0.0001*	1.0000	1.0000	0.0063*	0.9350	0.8785
*S. aureus* disc diffusion	2.4913	− 0.1875	0.0375	**1.875**	− 2.8031E-16	0.025	0.025	0.248913	0.023913	− 0.00108696
p-values		0.4806	0.8858	0.0001*	1.0000	0.9460	0.9460	0.3127	0.9197	0.9963
*E. coli disc* diffusion	2.80435	0.075	− 0.0125	**1.65**	− 1.73113E-16	0.05	− 2.53966E-16	− 0.0244565	− 0.0369565	− 0.199457
p-values		0.7627	0.9597	0.0002*	1.0000	0.8865	1.0000	0.9135	0.8697	0.3889
*S. entrica* disc diffusion	1.91739	0.075	− 5.35046E-17	**1.1**	− 4.35295E-17	0.05	-7.53195E-17	− 0.0228261	− 0.0228261	-0.147826
p-values		0.6707	1.0000	0.0003*	1.0000	0.8403	1.0000	0.8861	0.8861	0.3680
*S. cerevisiae* disc diffusion	3.68696	0.075	0.025	**2.55**	− 4.59964E-16	0.05	0.05	− 0.0391304	− 0.0391304	− 0.0391304
p-values		0.7911	0.9295	< 0.0001*	1.0000	0.9004	0.9004	0.8790	0.8790	0.8790

*Bold values denote statistical significance at the p < 0.01 level. ^a^UTS, ultimate tensile strength. ^b^SAB, strain at break. ^c^WVP, water vapor permeability. ^d^ΔE, color difference.

The data presented in Table 3 demonstrate the effectiveness of the model and its predictive capability for color difference and opacity properties across various formulations, thus confirming the efficiency of the experimental models for each response. The R^2^ coefficients were calculated to be 0.9932 and 0.9947, respectively. The final regression equations for the color difference (ΔE) response and opacity properties are expressed in the following equations:

14 $$\Delta E = 36.7741 - 16.8654X1 + 16.5308X2 + 23.4796X3 + 6.53811X1X2 - 10.1241X1X3 + 4.39417X_{1}^{2} + 4.15329X_{3}^{2}$$

15 $${\text{Opacity }} = { 1}.{65 } - \, 0.{3}00{\text{6X}}_{{1}} + \, 0.{45}0{\text{6X}}_{{2}} - \, 0.0{\text{294X}}_{{3}} + \, 0.{\text{3388X}}_{{1}} {\text{X}}_{{2}}$$

The disc diffusion test of the films in *S. enterica*, *S. cerevisiae*, *S. aureus*, and *E. coli* bacteria media utilized a quadratic model. The R^2^ coefficients for these tests were found to be 0.8367, 0.9244, 0.8598, and 0.8554, respectively, indicating a reasonably good level of correlation between the experimental values and the fitted values (Table 3). The final regression equations for the disc diffusion response are presented in the following equations:

16 $$S. \, enterica{\text{disc diffusion }}\left( {{\text{mm}}} \right) \, = { 1}.{73529 } + { 1}.{\text{1X}}_{{3}}$$

17 $$S. \, cerevisiae{\text{disc\; diffusion }}\left( {{\text{mm}}} \right) \, = { 3}.{57647 } + { 2}.{\text{55X}}_{{3}}$$

18 $$S. \, aureus{\text{disc diffusion }}\left( {{\text{mm}}} \right) \, = { 2}.{747}0{6 } + { 1}.{\text{875X3}}$$

19 $$E. \, coli{\text{disc diffusion }}\left( {{\text{mm}}} \right) \, = { 2}.{55882 } + { 1}.{\text{65X}}_{{3}}$$

### Influence of the effects on responses

#### Interaction effects of CMC, CHNF, and CMP on water sensitivity

**Figure 1:**
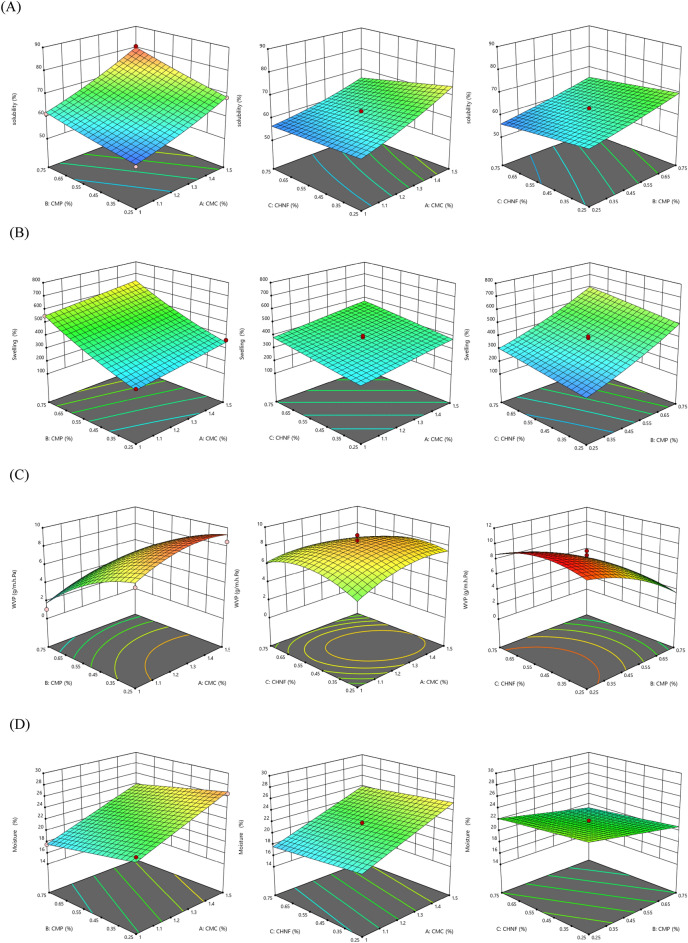
Surface plots for the effects of CMC, CMP, and CHNF in nanocomposite films on (**A**) Solubility (%), (**B**) Swelling (%), (**C**) WVP (g/mhPa) and (**D**) Moisture content (%).

In order to elucidate the relationships and interactions between the independent variables (CMC, CHNF, and CMP concentrations) and the responses (solubility, swelling, WVP, and moisture), 3D response surface plots were generated (Fig. 1). These plots depicted the variations in the responses as a function of the concentrations of the independent variables. Different shapes of the response surface plots were observed due to the various mutual effects among the variables. The significance of the independent variables on the responses is presented in Table 4.

As an essential factor, water solubility is a good indicator in evaluating the films’ resistance to water. This attribute becomes important when films encounter high-moisture food, especially during storage. preliminary studies before using RSM to find the best levels of the independent variable showed that CMC film dissolved partially, when encountering an aqueous medium ^25–27^. These initial findings underscore the importance of assessing water solubility as a key parameter in evaluating film performance and set the foundation for subsequent optimization efforts using RSM.

The linear and quadratic effects of CMC concentration (*X*_1_, ${X}_{1}^{2}$) were significant (P < 0.05) on the solubility. The interaction between CMC and other independent variables (X_1_X_2_, X_1_X_3_) also was significant (Table 4). The CMP concentrations showed linear and quadratic effects on the solubility (P < 0.01). However, the linear effects of CHNF concentrations (*X*_3_) on the swelling value of the films were significant (P < 0.01), and the quadratic effects were non-significant. These statistical analyses provide valuable insights into the individual and interactive effects of the independent variables on water solubility, guiding the formulation optimization process.

The mutual effects of the independent variables on the water solubility of the CMC-based films are depicted in Fig. 1A. Notably, the increase in CMC/CMP concentration had a significant effect, resulting in an increase in the solubility of the film across all concentrations of CMC and CMP. Additionally, the increase in CHNF concentration led to a decrease in the solubility of the CMC-based film, regardless of the concentrations of CMC and CMP. This can be attributed to the formation of a network structure by the well-dispersed nanofibers of chitosan, which interacted with the polymer and reduced the presence of free hydrophilic groups, consequently reducing the solubility 1. Similarly, previous studies have reported that the solubility of CMC-based films is reduced when blended with nano chitosan^3,28^. The graphical representation and mechanistic explanation elucidate the complex relationships between the independent variables and water solubility, enhancing the understanding of film behavior and informing potential applications.

The swelling percentages of the CMC-based films are depicted in Fig. 1B. The experimental findings revealed a high swelling value for the CMC-based films. The swelling ratio of the films was assessed after immersing the film samples in water for 24 h, and the swelling ratio increased with an increase in CMP and CHNF concentrations ranging from 0.25% to 0.75%. CMC is a water-soluble polymer that contains carboxyl and hydroxyl groups, imparting hydrophilicity to the molecule. The higher swelling rate can be attributed to the hydrophilic nature of CMC, which binds with CMP and CHNF. These results are consistent with the findings reported by Akhtar et al.^8^. Among all samples, the films containing 0.5% CHNF, 0% CMP, and 1.25% CMC exhibited the lowest swelling. The swelling behavior of CMC-based films provides valuable insights into their water absorption properties. The observed increase in swelling with higher concentrations of CMP and CHNF aligns with the expected behavior of hydrophilic polymers interacting with water molecules. The specific formulation resulting in the lowest swelling suggests a potential avenue for optimizing film properties for applications where reduced water absorption is desired.

The significance of the quadratic effects of independent variables (X_1_, X_2_, X_3_) and the linear effects of CMP (X_2_) on the water vapor permeability (WVP) of the films was confirmed (P < 0.01) (Table 4). Increasing the concentration of the independent variables led to a decrease in the WVP of the films (Fig. 1C). The improved water vapor barrier property (reduced WVP) of the composite films can be attributed to the presence of hydrophobic nanoparticles, which creates a convoluted path that hinders water vapor diffusion^27^. Additionally, the incorporation of CMP, CMC, and glycerol in the films promotes cross-linking, reducing the interactions between the polymer matrix and water molecules. As a result, water vapor molecules take longer to permeate through the films due to the increased thickness^8^. These findings are consistent with the results reported by other researchers^16,29^. The observed trends in water vapor permeability align with theoretical expectations, highlighting the role of nanoparticle incorporation and polymer cross-linking in enhancing barrier properties. The confirmation of significant effects from RSM underscores the importance of optimizing formulation parameters to achieve desired film properties for diverse applications.

Moisture content is a crucial factor in applying bio-based edible films^30^. The linear effects of CMC, CMP, and CHNF (X_1_, X_2_, X_3_) on the moisture content of the films were found to be significant (P < 0.05) (Table 4). The moisture content of the CMC-based films is illustrated in Fig. 1D. The experimental findings demonstrated a decrease in the swelling ratio with an increase in CMP and CHNF concentrations ranging from 0.25 to 0.75%. This suggests that polysaccharides may interact with CMC molecules through hydrogen bonding, leading to a reduction in the interaction between water molecules and the hydrophilic groups of CMC. Consequently, the binding of water molecules with CMC decreases, resulting in lower moisture content. Similar trends have been reported by other researchers^3^. The observed decrease in moisture content with increasing concentrations of CMP and CHNF provides valuable insights into the interaction mechanisms within the film matrix. This phenomenon, driven by potential hydrogen bonding interactions, contributes to the development of more stable bio-based edible films with reduced susceptibility to moisture, enhancing their suitability for various applications.

#### Mechanical properties

**Figure 2:**
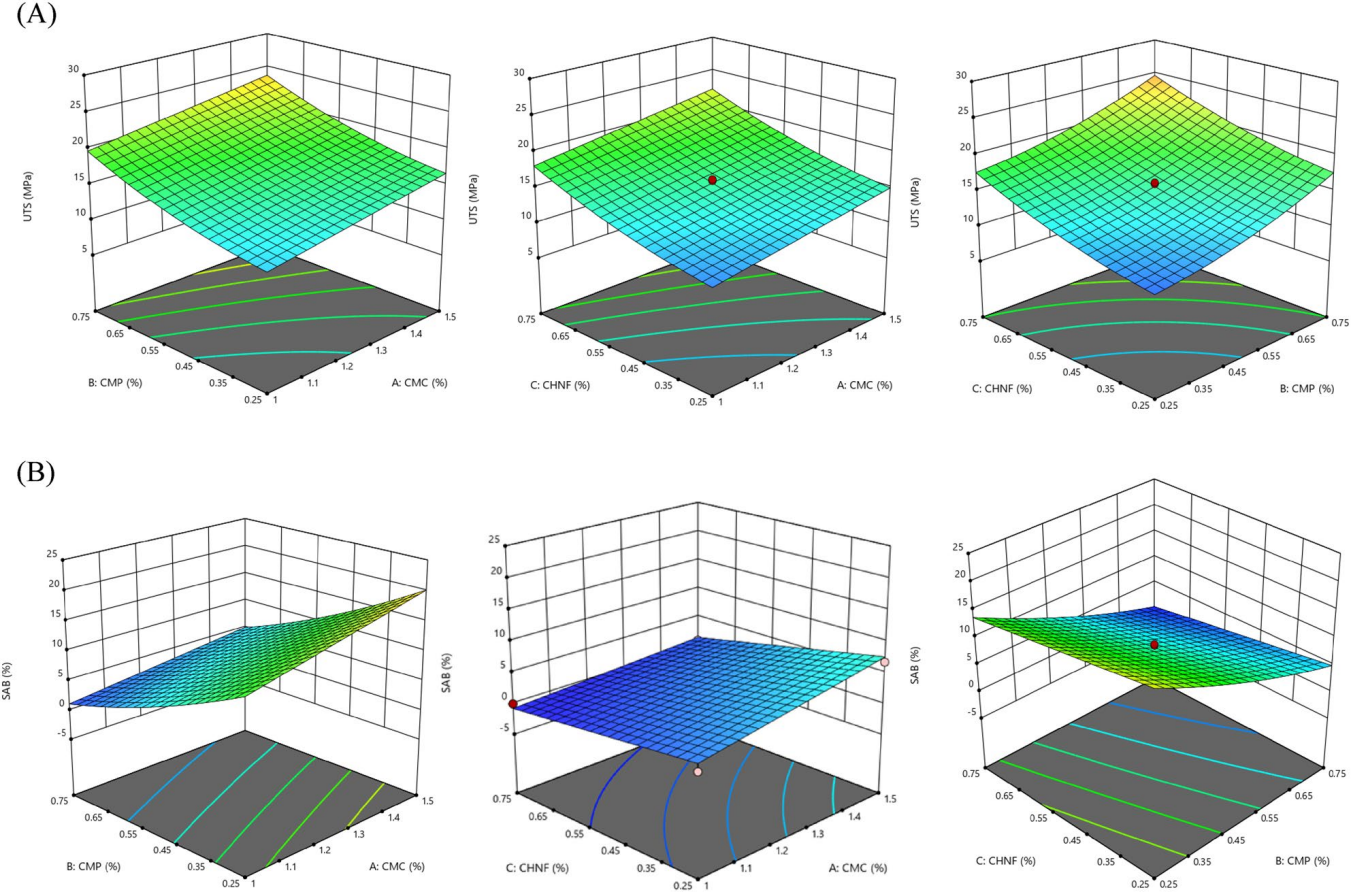
Surface plots for the effects of CMC, CMP, and CHNF in nanocomposite films on (**A**) UTS (MPa) and (**B**) SAB (%).

To investigate the impact of independent variables (CMC, CHNF, and CMP concentrations) and their interactions on the ultimate tensile strength (UTS) and strain at break (SAB), three-dimensional response surface plots were utilized, as shown in Fig. 2. The linear effects of CMC (X_1_), CMP (X_2_), and CHNF (X_3_) concentrations, as well as the quadratic effects of CMP (X_2c_^2^) and CHNF (X_3_^2^) concentrations, had a significant influence on the UTS value of the films (P < 0.01). However, the interaction effects were found to be non-significant (Table 4). Conversely, the linear effects of the independent variable concentrations, the quadratic effects of CMP (X_2_^2^), and the interaction effects between CMC and other independent variables (X_1_X_2_, X_1_X_3_) had a significant impact on the SAB value (P < 0.01). The utilization of three-dimensional response surface plots allows for a visual representation of the complex relationships between independent variables and film properties. While linear and quadratic effects were significant for UTS and SAB, the absence of significant interaction effects suggests that the individual contributions of the variables play a more dominant role in determining mechanical properties.

The ultimate tensile strength (UTS) of the films was enhanced by increasing the concentrations of CHNF and CMP, regardless of the CMC concentration (Fig. 2A, B). Conversely, the strain at break (SAB) value decreased with increasing CHNF and CMP concentrations, which can be attributed to the higher rigidity and lower flexibility of the CHNF/CMP/CMC complex compared to pure CMC network. The improvement in mechanical properties of the films may be attributed to the intermolecular interactions between the carboxyl group of CMC and the hydroxyl group of polysaccharide molecules. This could be a result of the strong intermolecular structure provided by CMC and CHNF, leading to increased mechanical properties. These findings are consistent with the results reported by Zhang et al.^31^, who demonstrated that the incorporation of chitosan and cellulose derivatives enhanced the strength of CMC-based films. The observed trends in UTS and SAB corroborate the mechanical enhancement achieved through the synergistic interactions between CMC, CHNF, and CMP. The literature reference further supports the validity of our findings and highlights the potential of incorporating biopolymer blends to improve film mechanical properties for various applications.

#### Color and opacity properties

**Figure 3:**
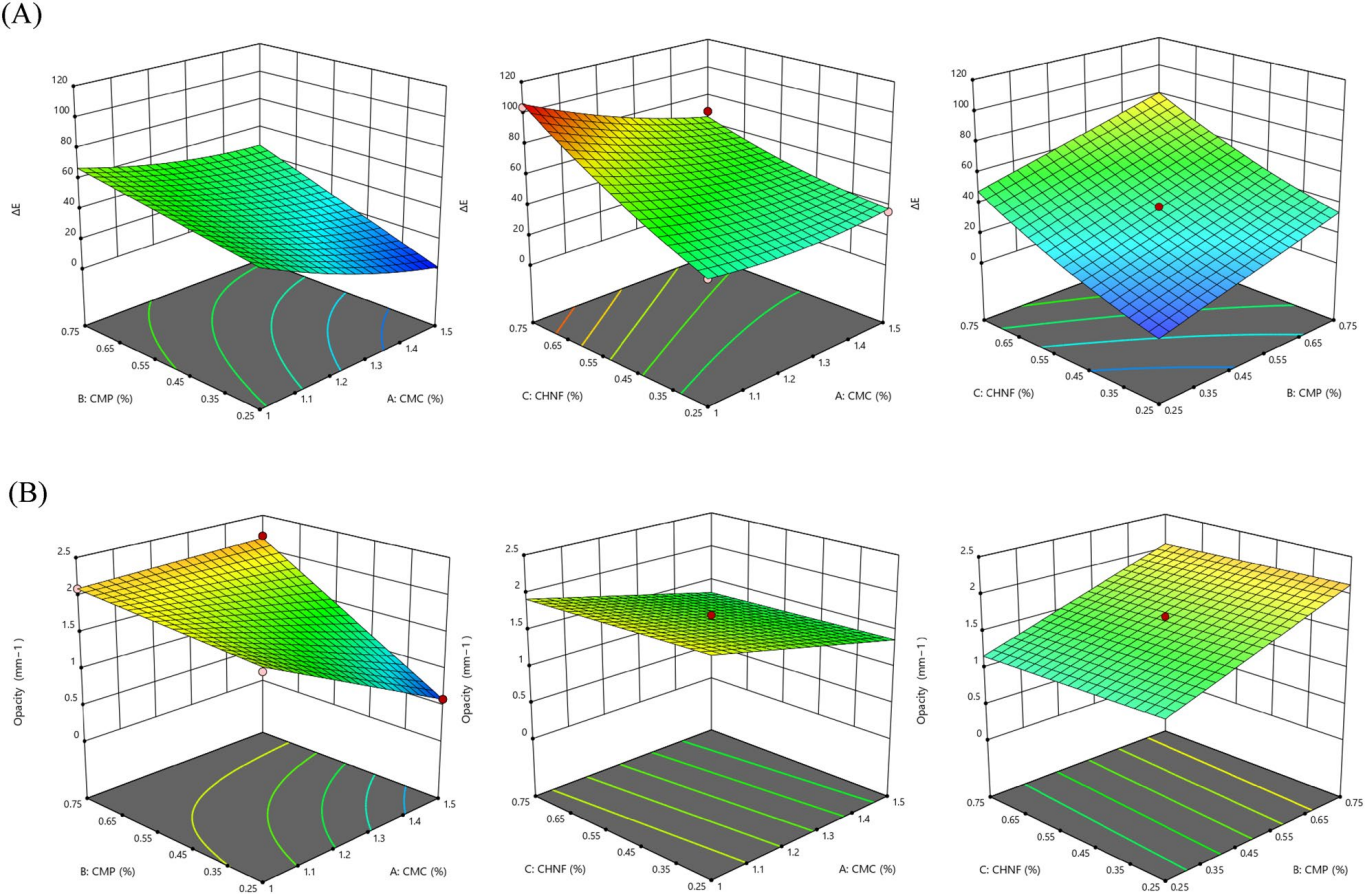
Surface plots for the effects of CMC, CMP, and CHNF in nanocomposite films on (**A**) ΔE factor, (**B**) opacity.

The color of films plays a crucial role in determining the acceptability of final food products among consumers. The linear and interaction effects of CMP (X_2_, X_1_X_2_) and CHNF (X_3_, X_1_X_3_) concentrations on the color difference (ΔE) value were found to be significant (P < 0.01). Additionally, the quadratic effects of CHNF (${\text{X}}_{3}^{2}$) were also significant (Table 4). To visualize the combined effect of these variables on the ΔE value of the nanocomposite films, 3D response surface plots were generated and presented in Fig. 3. It was observed that increasing the concentration of CMC led to a decrease in the ΔE value, while increasing the concentrations of CMP and CHNF resulted in an increased in the ΔE value. This finding aligns with the results reported by Azarifar et al.^2^, who observed a significant difference in the total color difference (ΔE) between gelatin-CMC based films and gelatin-CMC-CHNF nanocomposite films (p < 0.05), with the difference being influenced by the concentration of CHNF. Similarly, Akhtar et al.^8^ and Bandyopadhyay et al.^32^ reported similar results when polysaccharides were added to CMC films, noting evident changes in the color of the films upon the addition of gums. The films transitioned from being light and transparent (without gum) to having a brownish hue (gum-CMC film). The significant effects of CMP and CHNF concentrations on color difference underscore the importance of optimizing film formulations to meet aesthetic requirements. The literature references provide further support for our findings, emphasizing the impact of biopolymer additives on film color and consumer acceptability.

The linear and interaction effects of CMP (X_2_, X_1_X_2_) concentrations on the opacity value were found to be significant (P < 0.01). Transmittance and opacity are two contrasting factors representing the ratio of light transmission and light absorption in a material. Transmittance is associated with the alignment of polymers in the film structure. The control CMC film exhibited high transparency compared to the CMC-CMP-CHNF films. The transmittance decreased as the CMP concentration increased, which is consistent with previous studies by Akhtar et al.^8^ and Bandyopadhyay et al.^32^ that reported a similar decrease in transmittance in composite films. In this study, the decrease in transmittance can be attributed to the intermolecular bonding formed by the CMC and CMP networks. The results indicate that as the CMP concentration increased, the films became darker and brownish with lower transmittance, which could be attributed to the color of CMP. This can help protect packaged food from oxidative deterioration caused by UV radiation, which can lead to discoloration, nutrient loss, and the production of off-flavors. The significant influence of CMP concentration on film opacity highlights its potential role in providing UV protection to packaged foods. Understanding these effects contributes to the development of packaging materials that enhance product shelf life and quality.

#### Antimicrobial activity of films

**Figure 4:**
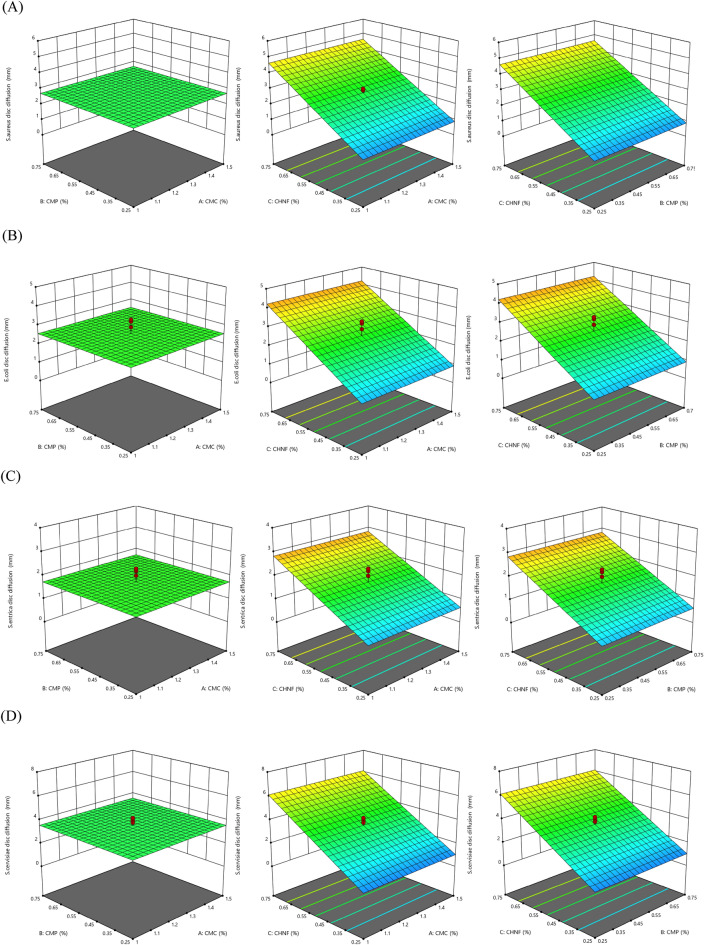
Surface plots for the effects of CMC, CHNF and CMP in composite films on (**A**) *S. aureus* disc diffusion (mm), (**B**) *E. coli* disc diffusion (mm), (**C**) *S. enterica* disc diffusion (mm) and (**D**) *S. cerevisiae* disc diffusion (mm).

The CMC and CMP concentration had no significant effect on the inhibitory zones for *S. aureus*, *S. entrica*, *E. coli*, and *S. cerevisiae*. The linear effect of CHNF (X_2_) on the inhibitory zone of all microorganisms was significant (P < 0.01) (Table 4). Furthermore, Based on Fig. 4, the CMC-based edible film, at all concentrations of CMC and CMP, and low concentrations of CHNF, showed no inhibitory zone against any of the microorganisms. However, increasing the concentration of CHNF led to an increase in the inhibitory zones against all microorganisms at higher CHNF concentrations. Based on Fig. 4, the antimicrobial films were more effective against *S. aureus* and *S. cerevisiae* compared to *E. coli* and *S. enterica*, possibly due to the single-layer cell wall of *S. aureus*, which makes it more susceptible than other bacteria like *E. coli*. These findings are consistent with the results of Qin et al.^33^ and Jannatyha et al.^3^, which demonstrated the vigorous antimicrobial activity of nano chitosan in nanocomposite films against gram-positive bacteria. While CMC and CMP concentrations did not significantly influence the inhibitory zones, the significant linear effect of CHNF concentration on antimicrobial activity highlights its potential as an antimicrobial agent in edible films. The observations regarding the efficacy against different microorganisms provide valuable insights into the potential applications and limitations of the antimicrobial films. The cited literature further supports our findings, reinforcing the robust antimicrobial activity of nano chitosan in similar film systems.

### General optimization of variables for CMC-based films

The statistical analysis using RSM yielded fitted models that allowed for the determination of the optimal concentrations of CMC, CMP, and CHNF based on the physical, mechanical, and antibacterial properties of the films, as presented in Table 2. To determine the optimal levels of the variables in general, numerical and graphical optimization procedures were employed, resulting in 46 suggested solutions by the software. Taking into account all parameters, the optimized variables were determined as 1.5 (w/v%) for CMC, 0.25 (wt%) for CMP, and 0.75 (wt%) for CHNF, resulting in an overall desirability of 66%.

### Experimental verification

To confirm the accuracy of the model equations, experimental verification was performed using the optimal concentrations. The results of both the experimental and predicted data for the responses at the optimum point are presented in Table 5. It can be observed that the experimental response values closely matched the predicted values, with only a small percentage of error between them. This indicates that the response surface equations adequately represent the experimental responses. The verification experiments validate the reliability of the model equations for the studied responses.

**Table 5:** Predicted and experimental data for the responses at the optimum point.

Analysis	Predicted data	Experimental data	Percentage error
UTS^a^	19.6109	18.35	6.87
SAB^b^	16.4038	16.4	0.01
Opacity	0.530037	0.493333	7.44
WVP^c^	7.37176	7.77	5.12
ΔE^d^	18.7954	18.5	1.59
Solubility	60.7837	60.8767	0.15
Moisture	25.4031	25.3667	0.14
Swelling	349.21	348.873	0.09
Thickness	0.0264524	0.0266667	0.8
*S. aureus* disc diffusion	4.62206	3.8	21.63
*E. coli* disc diffusion	4.20882	4.13333	1.82
*S. entrica* disc diffusion	2.83529	2.93333	3.34
*S. cerevisiae* disc diffusion	6.12647	5.16667	18.57

^a^UTS, ultimate tensile strength. ^b^SAB, strain at break. ^c^WVP, water vapor permeability. ^d^ΔE, color difference.

### Scanning *electron* microscopy (SEM)

**Figure 5:**
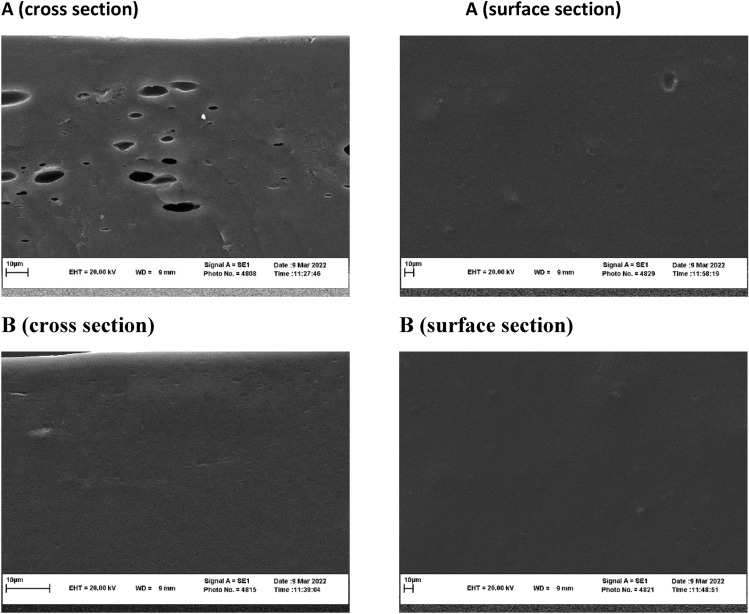
SEM images of prepared films: (**A**) CMC: 1.5 wt%., (**B**) CMC1.5 wt%./CMP: 0.25 wt% / CHNF: 0.75 wt%.

The SEM images of the films are depicted in Fig. 5A–B. The CMC film (Fig. 5A) showed a porous structure with some pores throughout its polymer matrix. However, the porosity of the film's structure decreased after the addition of CMP (0.25 wt%) and CHNF (0.75 wt%). It could be related to the presence of both of them in the film matrix, which replaced the weak interactions (non-covalent interactions) between CMC polymer networks with the strong interactions (covalent interactions) between CMC polymer matrix and CMP and CHNF, and consequently, produced a film with greater integrity, as already shown by the mechanical results (Fig. 2). The SEM images clearly explained the role of CMP and CHNF in improving the mechanical and barrier properties of the films (Fig. 5B). Similar observation was reported by Azarifara et al.^2^ who worked on the gelatin-CMC based active films containing chitin nanofiber. The SEM images provide valuable insights into the microstructure of the films, highlighting the changes induced by the incorporation of CMP and CHNF. The observed decrease in porosity and enhanced integrity of the film structure supports the mechanical and barrier property improvements observed in the mechanical results. This structural reinforcement is attributed to the strong interactions between CMC polymer matrix and CMP and CHNF, as evidenced by the SEM images. The reference to the work of Azarifara et al. (2019) further supports the validity of our findings, emphasizing the role of nanofiber additives in enhancing film properties.

### FTIR analysis

**Figure 6:**
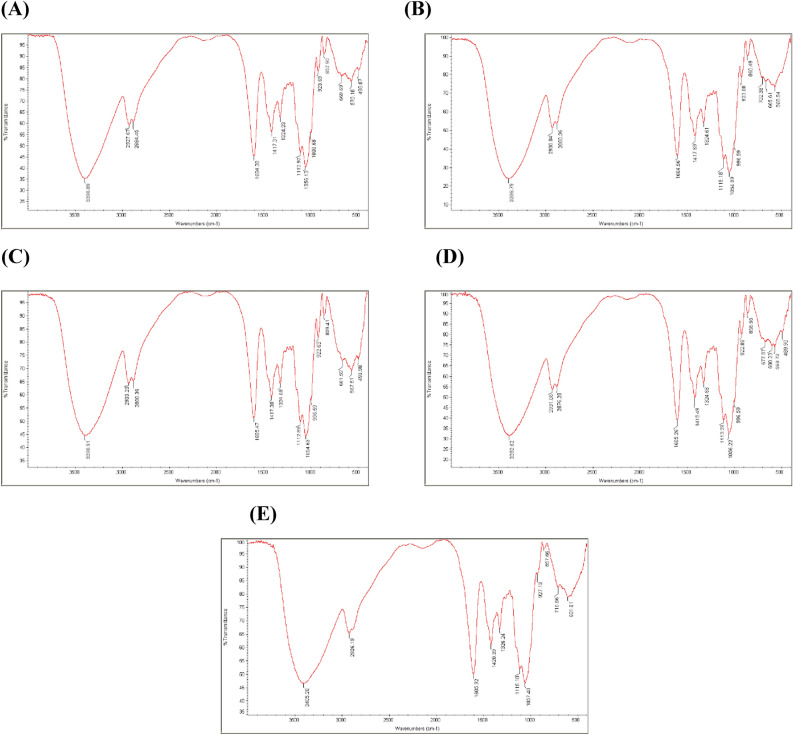
FTIR images of prepared films: (**A**) CMC:1.25 wt%./CMP: 0.5 wt%, (**B**) CMC: 1.25 wt%./CMP: 0.5 wt% / CHNF: 0.5 wt%., (**C**) CMC: 1.75 wt%./CMP: 0.5 wt% / CHNF: 0.5 wt%., (**D**) CMC: 1 wt%./CMP: 0.75 wt% / CHNF: 0.25 wt%., (**E**) CMC: 1 wt%./CMP: 0.75 wt% / CHNF: 0.25 wt%.

The FTIR images of the films are depicted in Fig. 6A–E. The FTIR images of the films with varying compositions of CMC, CMP, and CHNF show differences in the intensity and position of peaks, indicating changes in chemical bonding and interactions. Analyzing the FTIR spectra can provide insights into the functional groups present, molecular structure, and potential interactions between the components. The chemical interactions between CMC, CMP, and CHNF can contribute to the overall properties of the films by influencing the film's mechanical strength, thermal stability, and barrier properties, as observed in the FTIR images through changes in peak shapes and positions.

Comparative analysis of FTIR spectra for each of the films reveals significant differences in chemical properties and molecular structures. For instance, in the film with the composition CMC: 1.25 wt%./CMP: 0.5 wt%, peaks corresponding to carboxylic acid groups and hydroxyl groups in the range of 3000–3600 cm^-1^ are observable, indicating the presence of CMC and CMP. The addition of CHNF to the composition enhances the intensity of peaks related to hydroxyl and carboxylic acid groups, indicating chemical interactions among different components of the film. These differences highlight key points where variations in the composition of different materials have a significant impact on the molecular structure and properties of the films*.*

## Conclusion

The primary objective of this study was to investigate the impact of CMP and CHNF on the properties of biodegradable CMC-based films. Mathematical models were developed to describe the solubility, swelling, water vapor permeability (WVP), moisture content, mechanical properties, color, and antimicrobial characteristics of the CMC nanocomposite films based on varying concentrations of CMP and CHNF. The analysis of variance revealed that the developed models for all 13 responses exhibited significant effects (P < 0.01). The statistical performance and validation parameters confirmed the suitability of the models for predictive purposes. The quadratic models for solubility, swelling, WVP, strain at break (SAB), ultimate tensile strength (UTS), color difference (ΔE), and thickness, as well as the linear models for *S. aureus*, *S. entrica*, *E.coli*, *S. cerevisiae*, and the 2Fl model for moisture content, were found to be significant (P < 0.05). Furthermore, the results obtained from the verification experiments demonstrated good agreement between the experimental and predicted values, further supporting the reliability of the models.

The findings indicated that both CMP and CHNF had positive effects on reducing moisture content and water vapor permeability (WVP), as well as increasing ultimate tensile strength (UTS). However, higher concentrations of CMP and CHNF had an opposite effect on strain at break (SAB), the color difference (ΔE), and swelling. Incorporating CMP and increasing its concentration significantly increased opacity and solubility, while incorporating CHNF and increasing its concentration significantly decreased opacity and solubility. The decrease in solubility values of the CMC-based films can be attributed to the filling of empty spaces in the amorphous regions of the polymer matrix (as observed in SEM and FTIR images), leading to reduced chain mobility and flexibility.

Regarding the antimicrobial analysis, only the addition of CHNF noticeably improved the antibacterial properties of the resulting films. Furthermore, applying the optimization procedure (using RSM) to the biodegradable films containing CMC (1.5 wt%), CMP (0.25 wt%), and CHNF (0.75 wt%) yielded the best results in terms of physical, mechanical, and antibacterial properties. The comprehensive analysis conducted in this study demonstrates the effectiveness of CMP and CHNF as additives in improving various properties of CMC-based films. The successful development and validation of mathematical models using RSM highlight the predictive capabilities of the approach, offering valuable insights for future formulation optimization. The observed effects on film properties provide a deeper understanding of the interactions between polymer matrix and additives, contributing to the advancement of biodegradable film technology.

## Data Availability

The datasets generated during and/or analyzed during the current study are available from the corresponding author on reasonable request.

## Code availability

No code was attempted or used during the current manuscript.
